# NF-KappaB expression correlates with apoptosis and angiogenesis in clear cell renal cell carcinoma tissues

**DOI:** 10.1186/1756-9966-27-53

**Published:** 2008-10-19

**Authors:** Ibrahim Meteoglu, Ibrahim H Erdogdu, Nezih Meydan, Muhan Erkus, Sabri Barutca

**Affiliations:** 1 Adnan Menderes University, Medical Faculty, Department of Pathology, 09100-Aydin, Turkey; 2 Adnan Menderes University, Medical Faculty, Department of Medical Oncology, 09100-Aydin, Turkey

## Abstract

**Background:**

Clear cell renal cell carcinoma (ccRCC) is the most frequently encountered tumor in the adult kidney. Many factors are known to take part in the development and progression of this tumor. Nuclear factor kappa B (NF-κB) is a family of the genes that includes five members acting in events such as inflammation and apoptosis. In this study, the role of NF-κB (p50 subunit) in ccRCC and its relation to angiogenesis and apoptosis were investigated.

**Methods:**

Formalin-fixed and paraffin embedded tissue blocks from 40 patients with ccRCC were studied. Expressions of NF-κB (p50), VEGF, EGFR, bc1-2 and p53 were detected immunohistochemically. The relationship of NF-κB with these markers and clinicopathological findings were evaluated.

**Results:**

The expression of NF-κB was detected in 35 (85%), VEGF in 37 (92.5%), EGFR in 38 (95%), bc1-2 in 33 (82.5%) and p53 in 13 (32.5%) of 40 ccRCC patients. Statistical analyses revealed a significant relation between NF-κB expression and VEGF (p = 0.001), EGFR (p = 0.004), bc1-2 (p = 0.010) and p53 (p = 0.037). There was no significant correlation between NF-κB and such parameters as tumor grade, stage, age and sex.

**Conclusion:**

The results of this study indicated that in ccRCC cases NF-κB was associated with markers of angiogenesis and apoptosis such as VEGF, EGFR, bc1-2 and p53. In addition, the results did not only suggest a close relationship between NF-κB and VEGF, EGFR, bc1-2 and p53 in ccRCC, but also indicate that NF-κB was a potential therapeutic target in the treatment of ccRCC resistant to chemotherapy.

## Background

Renal cell carcinoma (RCC) is the most common malignant tumor of the adult kidney, accounting for 3% of all adult malignancies [1]. Clear cell renal cell carcinoma (ccRCC) accounts for approximately 70% to 80% of all cases of RCC [1,2]. In about two thirds of the patients with RCC, the disease is localized. Localized disease can be treated with radical or partial nephrectomy, and a vast majority is cured with surgery, but the prognosis is poor once the disease advances. The therapeutic options for such patients are few because chemotherapy is ineffective [2]. Nuclear factor kappa B (NF-κB) is a family of transcription factors, the functions of which are very well known in apoptosis and inflammation [3,4]. In normal cells NF-κB activation is regulated tightly. Normally, NF-κB can be activated by an appropriate stimulus and returns to the inactive status after the transcription in the target genes have been completed. In tumoral cells, different types of molecular alterations may result in damaged regulation of NF-κB activation. The resulting alterations participate in the development and progression of cancer [5-10]. Previous studies have shown the role of NF-κB in hematological or solid (like lung, gastric and prostate) tumors [11-17]. There is evidence for the role of NF-κB in RCC. Oya et al. demonstrated that increased NF-κB activity is associated with the development and progression of RCC [18]. Vascular endothelial growth factor (VEGF) is a strong stimulator of the mitogenic activity in the endothelial cells. There have been many studies demonstrating the prognostic importance of angiogenesis in various solid tumors [19]. High VEGF expression, increased microvascular density and advanced stage in RCCs usually indicate a poor prognosis [20-24]. Numerous studies have shown the association of EGFR with tumor proliferation, invasiveness and angiogenesis [25]. EGFR, which indicates a poor prognosis in many tumors, has also been shown to play a role in the development and progression of RCCs [26]. Furthermore, high EGFR levels are frequently associated with high grade tumors, indicative of a poor prognosis [26]. Tumor suppressor gene, p53, helps to repair damage in the DNA during the cell cycle and/or to direct the cell to apoptosis. As a result of mutations in the p53 gene, responsible for the development of many tumors, damaged cells may escape from apoptosis. In RCCs, high p53 levels are associated with an advanced tumor and poor prognosis [27,28]. bc1-2 is an intracellular membrane protein which is well known to play a role in the inhibition of apoptosis in many types of cancer. It is commonly thought that bc1-2, which has an antiapoptotic activity, may be effective in increased resistance to chemotherapy in advanced stage RCCs [27-29]. There is little known about the association of NF-κB with RCCs [30].

The aim of this study was to investigate the relation between the p50 subunit of NF-κB and the factors like apoptosis and angiogenesis, which affect tumor development and progression in the frequently encountered adult renal tumors, ccRCCs. Detection of a relation between important growth factors and various genes like VEGF, EGFR, p53 and bc1-2 and NF-κB activation will help to enhance the effectiveness of the available therapies and to develop new treatment modalities in the course of curative efforts for RCCs, which are considered as chemoresistant tumors [31,32]. NF-κB is likely to be incorporated into routine pathological investigations for RCCs and some other tumors in the future. Another aim of this study was to provide support for the use and standardization of immunohistochemical techniques widely used to investigate NF-κB status either in clinical practice or for research in the pathology laboratories.

## Methods

### Patients

Forty patients who underwent radical or partial nephrectomy for ccRCC at Adnan Menderes University Hospital from 2002 to 2007 were included in the study. Data about sex, age, tumor size, TNM stage and Fuhrman's nuclear grade were obtained. None of the patients received treatment for cancer before surgery. All specimens were re-evaluated before the current study by two pathologists. Paraffin-embedded specimens that contained normal renal tissue together with the tumoral tissue were selected for immunohistochemical analysis.

### Immunohistochemistry

First, 4 μm thick slices taken from the selected paraffin embedded blocks were placed on positively charged slides and deparafinized with xylene and rehydrated with ethanol. Second, all were washed with 0.1% Tris-buffered saline (TBS) three times for 5 minutes. They were kept in 0.3% hydrogen peroxidase for 20 minutes for the blockage of endogenous peroxidase activity. Third, the slices were rinsed with TBS three times for 5 minutes. For antigen retrieval, the slides were placed in a sodium citrate buffer and left in the microwave oven at 700-W for 10 minutes. Fourth, they were cooled to room temperature and washed with TBS three times for 5 minutes. Fifth, non-specific binding was blocked with 10% normal rabbit serum for 1 hour. Sixth, the slices were incubated with primary antibodies rabbit polyclonal NF-κB/p50 (1:50 dilution, RB-1648; NeoMarkers, Fremont, CA, USA), VEGF (ready solution for use, RB-9031; NeoMarkers, Fremont, CA, USA), EGFR (ready solution for use, MS-378; NeoMarkers, Fremont, CA, USA), p53 (ready solution for use, MS-738; NeoMarkers, Fremont, CA, USA) and bcl-2 (ready solution for use, MS-123; NeoMarkers, Fremont, CA, USA) for 1 hour. After the slides were rinsed with TBS three times for 5 minutes, they were exposed to biotin free horseradish peroxidase (HRP) enzyme-labeled polymer (EnVision plus detection system, ChemMateTM EnVision +/HRP Rb&Mo, DAKO, Hamburg, Germany) for 30 minutes. Seventh, the slides were rinsed again in TBS three times for 5 minutes. Eight, 3,3'-diaminobenzidine (DAKO) chromogen substrate was added. Last, the slides were rinsed under tap water and countered with Mayer's haematoxyline, dehydrated and mounted. For the negative control, the slides were incubated with TBS without the primary antibody. The slides known to be positively immunostained were used as positive controls.

### Evaluation of immunostaining

Firstly, the slides were examined at low magnification (×100) and areas containing the highest density of stained markers were chosen as "hot spot" areas and were evaluated at high magnification (×200). The staining cell ratio was determined by counting at least 200 cells. A staining percentage of 10% or above was accepted to be positive for all the markers. The scoring was accomplished according to the degree of staining in different magnifications as in the following: "weak" staining, visible at × 200; "moderate" staining, visible at × 100 and "strong" staining visible at × 40.

### Statistical analysis

Chi-square test for trend was used to assess the differential expression of markers in malignant and non-malignant kidney tissues. Spearman's rank correlation coefficient was used to determine the correlation between ink patterns of markers in RCC. P < 0.05 was considered significant. All statistical analyses were performed with SPSS (Windows version 13.0, SPSS Inc. Chicago, IL, USA).

## Results

Clinicopathologic characteristics of the patients are summarized in Table 1.

**Table 1:** Patients characteristics (n = 40)

	**n (%)**
Age (year)	
Range	35–85
Median	63
Sex	
Male	30 (75)
Female	10 (25)
Tumor size (cm)	
Range	1–20
Median	5.75
T stage	
T1	21 (52.5)
T2	11 (27.5)
T3	7 (17.5)
T4	1 (2.5)
Fuhrmans' Grade	
G1	13 (32.5)
G2	20 (50)
G3	7 (17.5)

**Figure 1:**
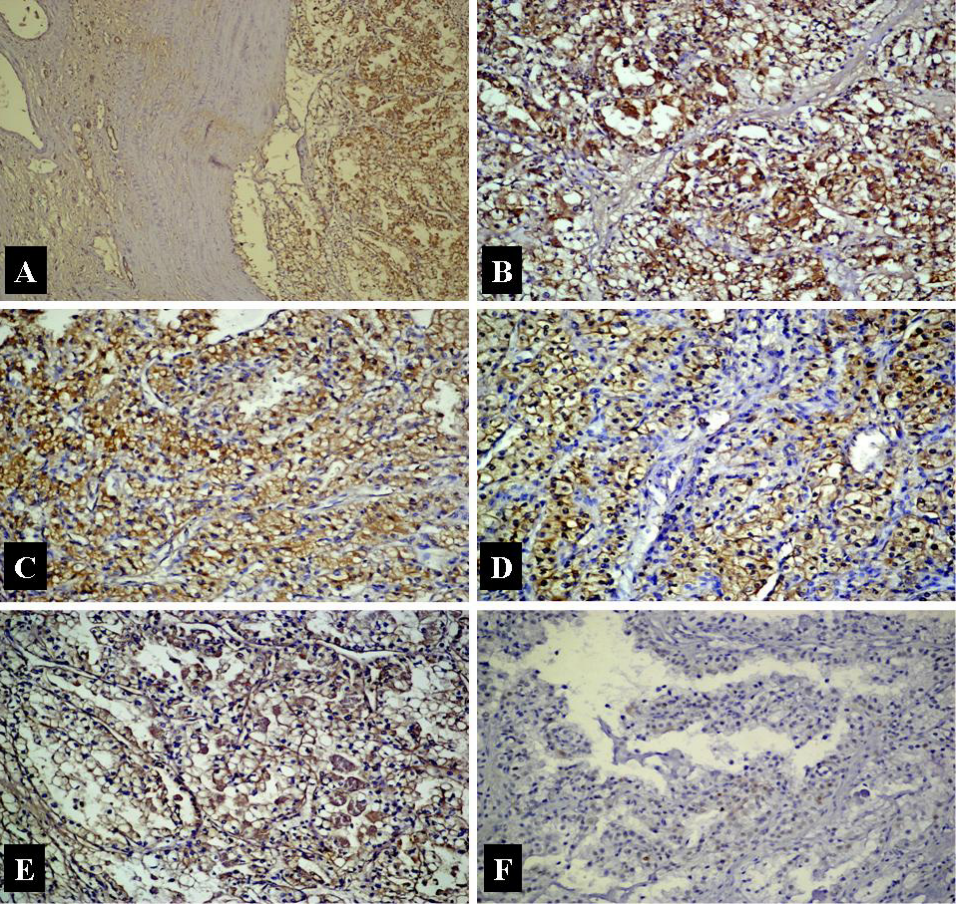
**Strong immunostaining for markers in ccRCC.** Expression of NF-κB in malignant and non-malignant renal tissue (A), high-level immunstaining for NF-κB (B), VEGF (C), EGFR (D), bcl-2 (E), and p53 (F). Note that increased NF-κB expression is associated with increased VEGF, EGFR, bcl-2 and p53.

**Figure 2:**
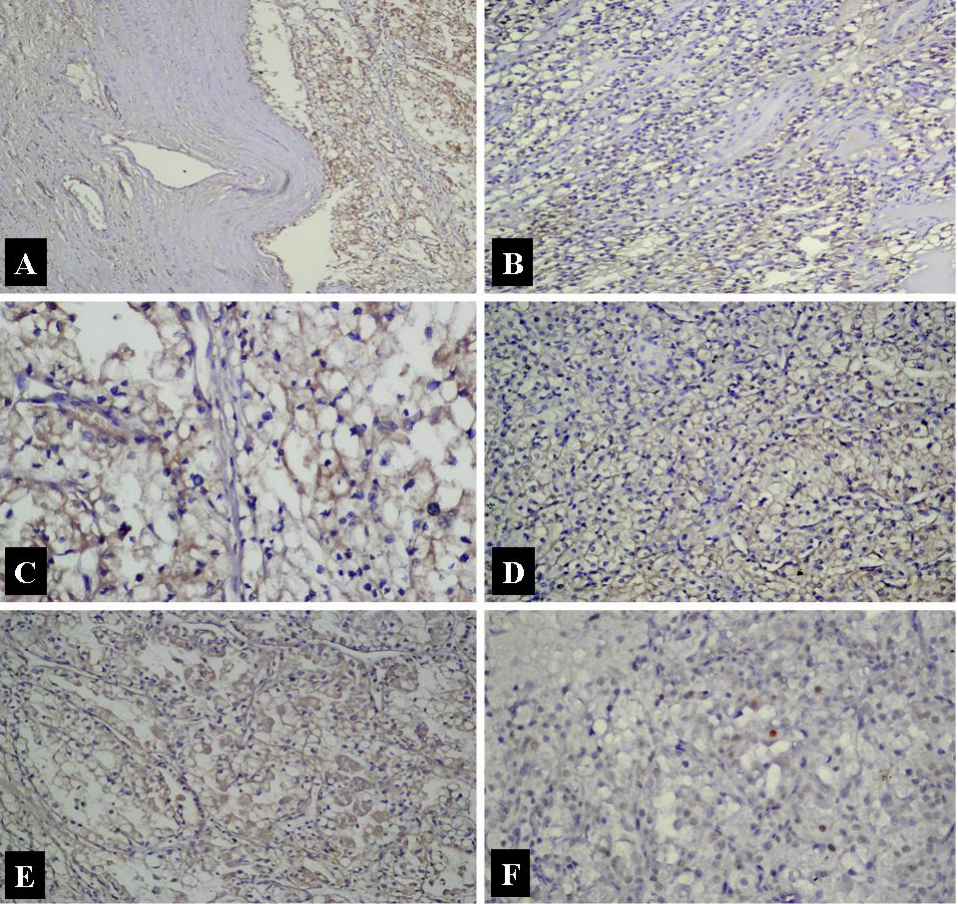
**Weak positive immunostaining for markers in ccRCC.** Expression of NF-κB in malignant and non-malignant renal tissue (A), low degree of immunostaning for NF-κB (B), VEGF (C), EGFR (D), bcl-2 (E), and p53 (F).

NF-κB staining was detected within the cytoplasm and nuclei of the tumoral cells. The degree of nuclear staining decreased towards the central areas of the tumor whereas the most prominent nuclear staining was seen in the areas adjacent to the non-tumoral renal tissue (Figure 1A). There was prominent cytoplasmic and nuclear staining within the histiocytes in the tumoral area (Figure 1B). While various degrees of staining were detected in 34 patients (85%) with NF-κB, there was no staining in 6 patients (15%). A strong (+++) positive staining was present in 19 patients. The staining was moderate in 10 patients and weak in 5 patients. The majority of the tumors that showed a weak staining were stage I tumors (Figure 2A–B). In 22 patients there was also a weak cytoplasmic staining in the tubular cells of the non-tumoral renal cells (Figure 2A). While there was no glomerular staining, there was some background staining within the interstitial areas.

VEGF staining was present within the cytoplasm of the tumor cells (Figure 1C, 2C). The staining degree was weak to moderate in most of the patients. Staining was present in 37 patients (92.5%), but there was none in 3 patients (7.5%). In 18 patients, a weak staininig with VEGF was present in tubules of the non-tumoral renal tissues. No glomerular or interstitial staining was detected. The only positive correlation was between VEGF and NF-κB. The tumour cell membranes were stained with EGFR. In 6 patients tubular cells of the non-tumoral tissue were also stained. No glomerular and interstitial staining was present. There was staining with EGFR in 38 (95%) patients, whereas no staining was present in 2 (5%) patients. The staining was moderate in the majority of the patients (Figure 1D, 2D). Apart from NF-κB, EGFR staining was also positively correlated with p53 (r = 0,370, p = 0.034). There were no other significant correlations with any other parameters. We found cytoplasmic staining with bc1-2 (Figure 1E, 2E). While there was no staining in 7 patients (17.5%), 33 patients (82.5%) displayed a positive staining. The degree of staining was predominantly moderate. In 34 patients we detected staining in the non-tumoral spaces. It was marked within the tubular epithelial and interstitial inflammatory cells. p53 staining was found to be within the nuclei of the tumor cells (Figure 1F, 2F). Thirteen patients (32.5%) displayed staining, whereas 27 patients (67.5%) showed no staining. The degree of the staining was predominantly weak. There was also some staining within the tubular epithelia in the non-tumoral area. There was neither glomerular nor interstitial staining.

Immunohistochemical staining properties of NF-κB, EGFR, VEGF, bcl-2 and p53 are shown in Table 2.

**Table 2:** Staining Properties of NF-κB p50 subunit, EGFR, VEGF, bcl-2 and p53 in ccRCC

	**Negative**	**Weak**	**Moderate**	**Strong**
**NF-κB**	6 (15%)	5 (12.5%)	10 (25%)	19 (47.5%)
**VEGF**	3 (7.5%)	12 (30%)	14 (35%)	11 (27.5%)
**EGFR**	2 (5%)	9 (22.5%)	20 (50%)	9 (22.5%)
**bcl-2**	7 (17.5%)	11 (27.5%)	16 (40%)	6 (15%)
**p53**	27 (67.5%)	7 (17.5%)	4 (10%)	2 (5%)

There was a significant positive correlation between the staining degree of NF-κB and VEGF (r = 0,516, p = 0,001), EGFR (r = 0,448, p = 0,004), bcl-2 (r = 0,403, p = 0,010) and p53 (r = 0,332, p = 0,037). No correlation between the NF-κB staining degree and age, sex, tumor size, TNM stage and Fuhrmans' grade was established.

## Discussion

NF-κB activation plays a role in the regulation of more than 400 gene product expressions associated with inflammation, cell survival, proliferation, invasion and angiogenesis [3,7]. The NF-κB family contains five genes, which are NF-κB1 (p59/p105), NF-κB2 (p52/p100), Re1A (p65), c-Rel and RelB. The NF-κB dimers have been shown to be present in the cytoplasm of many cells in an inactive state. NF-κB activation can be initiated by the stimuli of various signaling pathways like cytokines, growth factors and tyrosine kinases [4]. Various molecular mechanisms have been described for the activation of NF-κB. While the classical or canonical NF-κB pathway involves the Re1A or the c-REL dimers together with p50, the alternative pathway involves Re1B and p100 subunits. Tumor cells have such physiology that they can grow rapidly, avoid apoptosis, are irresponsive to growth-inhibitory signals and have capability of angiogenesis, invasion and metastasis and an infinite replicative potential. Almost all of the genes involved in the regulation of these processes are under the control of NF-κB transcription. The role of NF-κB in hematological and solid tumors has been described previously in many studies [7]. It has been shown that there is a relation between the development and progression of solid tumors and the subunits of NF-κB [8]. Many studies have revealed a relation between c-REL subunit and development of breast cancer and non-small cell lung cancer and the role of the p50–p65 subunits of NF-κB in the carcinogenesis of the breast [12,14,15]. Furthermore, an association between the NF-κB activation (Bc1-3/p50 complex) and development of nasopharyngeal carcinoma has been demonstrated [33]. Nair et al. have shown an increased NF-κB nuclear immunostaining in cases of cervical squamous cell carcinoma and in the presence of p65-p50 activation [17]. Oya et al. demonstrated the role of increased NF-κB activation in the development of RCC [18]. According to the results of their study, both the p65 and the p50 subunits of NF-κB have an increased activation in RCC cases. Most of the studies concerning the association between NF-κB and tumor development usually involves the p65 subunit. In the present study, the p50 subunit had an increased activation in ccRCCs, which supports the findings from the literature that NF-κB has an impact in some different ways on ccRCCs. In addition, there was a significant relation between increased NF-κB activity and increased bc1-2, p53, EGFR and VEGF activities.

NF-κB has an effect on apoptosis during tumor development and progression in many ways [34]. However, recent studies have yielded conflicting results about the effect of NF-κB and its interaction with apoptosis [7]. Some purport that NF-κB have a dual effect on apoptosis hence acting as an inhibitor or an activator depending on the levels of the p65 and cRel subunits [7]. However, the commonly accepted view is that the activation of NF-κB leads to apoptosis resistance. One of the pathways that NF-κB is effective in the inhibition of apoptosis is the bc1-2 gene family, which has an antiapoptotic effect. A few studies conducted on RCCs have shown a role of bc1-2 increased activity in resistance to chemotherapy in advanced stage tumors [35]. Morais et al. demonstrated decreased antiapoptotic bc1-2 and bcl-x protein levels in patients taking NF-κB inhibitors for metastatic RCCs, whereas there was not a significant change in proapoptotic bax protein levels [36]. We found a significant correlation between the increased NF-κB and bc1-2 expression levels. Our findings suggest that an increase in the NF-κB activity together with the bc1-2 activity plays a significant role in the inhibition of apoptosis. It has been reported that apoptosis is likely to be inhibited by NF-κB via p53 pathway. As in many other tumors, in RCCs, mutations that may take place in the p53 gene may impair the activity of NF-κB. There have been some studies describing the antiapoptotic activity of NF-κB by eliminating the wild type p53 dependent apoptosis [7,37]. In the present study, immunohistochemically, increased mutant p53 levels were significantly associated with an increased expression of NF-κB. This finding supports the idea that NF-κB prevents apoptosis through various pathways.

Angiogenesis plays a key role in the development and progression of tumors. Angiogenesis in tumors has been shown to be associated with the chemokines of the macrophages, neutrophils and other inflammatory cells (like monocyte chemoattractant protein, IL-8) and the growth factors (like TNF, VEGF). NF-κB activation plays a role in the regulation of these angiogenesis inducing products [4,7]. VEGF, especially, is a multifunctional growth factor family that has specific effects on endothelial cells. It undertakes an important role in tumor growth as in many other physiologic events. Increased VEGF activity in RCCs indicates a poor prognosis [22-24]. Various studies have demonstrated the inhibition of angiogenesis by the blockage of NF-κB [38]. Arai et al. reported the decline of the products like VEGF by the inhibition of NF-κB with dexamethasone administration in RCC cases [39]. The review of the literature revealed only a few studies that reveal an association of NF-κB with VEGF in RCCs. These studies have demonstrated that inhibition of NF-κB decreases VEGF levels [39,40]. We found a significant correlation between increased VEGF and NF-κB activity in RCCs, characterized by abundant neovascularization. Increased VEGF, shown to be an angiogenic factor in many neoplasms and considered as an indication of a poor prognosis, has been reported to be associated with increased NF-κB activation in RCCs, consistent with the result of the present study.

EGFR expression is frequently associated with proliferation, invasiveness and angiogenesis in many tumors and also considered an indicator for poor prognosis. It has been reported that the overexpression of the EGFR in RCCs plays a role in the development and progression of the tumor and is frequently accompanied by high grade tumors [41,42]. There have been a few studies showing an association between EGFR and NF-κB in RCCs. Some drugs used as EGFR inhibitors in RCCs act as maximum cytotoxic agents after NF-κB blockage. An et al. demonstrated that the signal transmission in the NF-κB activation via EGFR was performed by the phosphotidylinositol-3-OH kinase/AKT (PI3K/AKT) pathway [41]. They reported that blockage of NF-κB with EGFR inhibitors adds to the maximal cytotoxic effects of proteosome inhibitors. Similarly, the present study showed that increased NF-κB activity was associated with increased EGFR expression. In an effort to understand the reasons why there is a resistance to chemotherapy, especially for the advanced stage RCCs and to eliminate this problem, it is important to demonstrate the interaction of NF-κB with the genes like EGFR in order to be able to develop new modes of therapy [43-45].

In some studies exploring the association between the NF-κB activation and the tumors, a correlation between the prognostic indicators like tumor grade and stage and NF-κB have been demonstrated. Oya et al. found that NF-κB activity increased in advanced stage RCCs and that NF-κB played a role in the progression of the tumors at advanced stages [18]. In the present study, there was a stronger staining with NF-κB in the advanced staged ccRCC cases, but it was not statistically significant. Moreover, Fuhrmans' grade, which is an important prognostic indicator in RCCs, did not have a significant association with NF-κB activation, either. It may be that the patients we included in this study had mostly early stage tumors or that the sample size was small.

In this study we used immunohistochemistry, which is a simple reproducible method for the pathologists to conduct an investigation or use routinely in the investigation of or the estimation of NF-κB, VEGF and EGFR in the future. The review of the literature revealed only a couple of studies concerning the evaluation of NF-κB with immunohistochemical methods. NF-κB signaling pathway activation results in translocation of NF-κB subunit in the nucleus. Therefore, NF-κB activation can be identified by positive nuclear immunostaining of various NF-κB subunits. Nuclear immunostaining has been evaluated in many studies aiming to determine NF-κB subunits. Using immunochemical methods, Levidou et al. in their study on patients with gastric carcinoma evaluated both nuclear and cytoplasmic expression of NF-κB p50 subunit [16]. They found that nuclear immunostaining was associated with such parameters as stage and depth of invasion and an adverse effect on survival. They noted that cytoplasmic NF-κB immunostaining did not yield important prognostic information. Cogswell et al., using immunohistochemical staining, showed nuclear overexpression of p50, p52 and c-Rel subunits of NF-κB in human breast cancer [14]. They evaluated nuclear staining to show activation of NF-κB subunits. Lessard et al. in their study on patients with prostatic adenocarcinoma immunohistochemically showed increased expression of nuclear p50 of NF-κB [13]. Thornburg et al. revealed that nuclear expression of the p50 subunit of NF-κB played a role in the development of nasopharyngeal carcinoma [33]. In the present study, almost all patients had cytoplasmic staining with NF-κB in the tumoral tissues and nuclear staining was examined for the evaluation of NF-κB activation.

Recent studies have focused on new therapeutic agents and treatment alternatives with better antitumour effectiveness on advanced RCCs [46,47]. Angiogenesis is an extremely rare event in adults except for wound healing, inflammation and ischemic conditions. It is required that specific treatment alternatives targeting the angiogenetic pathway and having minimal toxicity should be sought. Vascularization in tumours can be inhibited by blocking endothelial growth factors with pseudo ligands, protein kinase inhibitors and monoclonal antibodies. Therefore, such factors as VEGF and VEGFR have become interesting targets of drug treatment for ccRCC. Clinical studies on Sunitinib (Sutent), Sorafenib (Nexavar) and mTOR inhibitors, small molecular VEGFR inhibitors used in advanced stage ccRCC, have rarely shown complete response to treatment [47]. Due to mechanisms which are still unclear, the patients receiving long-treatment become resistant to inhibition of neoangiogenesis [48,49]. With studies on tumour markers and development of treatment alternatives directed towards molecular targets, it seems that a new era for RCC treatment has started. Although these treatment alternatives have not achieved complete and long-term remissions, these genetic and molecular tumour markers allow detection of biological behaviour of each tumour and administration of specific treatments instead of non-specific treatments. The results of this study will contribute to development of new target molecules. However, further studies are needed to determine the role of various subunits of NF-κB in RCC and other tumours and to develop new treatment options.

## Conclusion

We found that NF-κB was associated with numerous factors in the development and progression of tumors in ccRCCs and that increased NF-κB activity was accompanied by increased bc1-2, p53, VEGF and EGFR expressions. To conclude, in cancer cells, NF-κB activity is correlated with events like proliferation, apoptosis, angiogenesis, chemo-radioresistance and has a diagnostic and prognostic importance. It is very important to develop new target molecules and to establish new chemotherapic strategies in malignancies like RCCs, which are resistant to chemotherapy. For the antiangiogenic and EGFR inhibitor drugs, which are lately being introduced to the therapeutic line in the treatment of many malignancies, knowing the effects and interactions of the molecules like NF-κB in order to be able to predict and increase their impact, is quite important. Moreover, NF-κB itself will be a target on its own for the chemotherapeutic agents. Therefore, we need to know the interaction of the various subunits of NF-κB in different tumors and their relation with different molecules. Establishing the subunits of NF-κB with the simple methods as we used in this study will enable new studies to be performed on the issue.

## Competing interests

The authors declare that they have no competing interests.

## Authors' contributions

IM conceived of the study and wrote the manuscript. IHE collected the samples and patient's clinical data. ME and NM participated in the design of the study and helped write the paper. SB reviewed the manuscript and wrote the final version. All authors read and approved the final manuscript.
